# Professor Andreas Retzius

**Published:** 1860-07

**Authors:** 


ANDREAS RETZIUS, Professor of Anatomy and Physiology in the Royal Caroline Institute of Stockholm, expired, after a short illness, on the 18th of April, in the sixty-fourth year of his age. Sweden has suffered in his death an irreparable loss, as an original investigator and a man of high scientific attainments. He was a forcible lecturer, always commanding the attention of his classes, and a very voluminous author, the titles of his scientific works alone filling three pages of the Biographical Dictionary.

